# Auditory-perceptual evaluation of voice: comparing different speech tasks to identify children with and without laryngeal lesions

**DOI:** 10.1590/2317-1782/20212021198en

**Published:** 2023-03-03

**Authors:** Cintia Conceição Costa, Ana Paula Dassie Leite, Glaucya Madazio, Mara Behlau

**Affiliations:** 1 Departamento de Fonoaudiologia, Universidade Estadual do Centro-Oeste – UNICENTRO - Irati (PR), Brasil.; 2 Centro de Estudos da Voz – CEV - São Paulo (SP), Brasil.

**Keywords:** Vocal Quality, Dysphonia, Children, Voice, Voice disorders

## Abstract

**Purpose:**

To compare the vowel emission and number counting tasks in perceptual-auditory differentiation among children with and without laryngeal lesions.

**Methods:**

Observational, analytical, and cross-sectional methods were used. Medical records of 44 children were selected from a database of an otorhinolaryngology service at a University Hospital and they were divided into groups: without laryngeal lesion (WOLL), and with laryngeal lesion (WLL), with 33 and 11 children. For the auditory-perceptual evaluation, the vocal samples were separated according to the type of task. They were analyzed separately by a judge who analyzed the general degree of vocal deviation and assessed whether the child would pass or fail in the face of a screening situation.

**Results:**

There was a difference between the WOLL and WLL groups in terms of the overall degree of vocal deviation for the task of number counting, with a predominance of mild deviations in WOLL and moderate in WLL. In the screening, there was a difference between the groups during the number counting task, with more failures in the WLL. The groups were similar in the sustained vowel task, both in terms of the overall degree of vocal deviation and the vocal screening. Most children in the WLL failed in both tasks during vocal screening compared to the children in the WOLL who, in general, failed in only one task.

**Conclusion:**

The task of number counting contributes to the auditory differentiation in children with and without laryngeal lesion, by identifying deviations of greater intensity in children with laryngeal lesion.

## INTRODUCTION

Vocal disorders during childhood can negatively affect the child's quality of life in relation to communication and participation in social, educational and group activities^(1)^. Although voice disorders in childhood are between 6% to 38%^(2,3)^, vocal symptoms are often neglected by family members, who do not think hoarseness is a problem, and value, to a much greater extent, only the speech disorders of children^(4)^.

There is a great complexity in the evaluation of children’s voices, mainly because their anatomo-physiological characteristics are very different from those of adults in relation to breathing (subglottic pressure higher than necessary for phonation, causing mismatch between systems), and laryngeal characteristics (immaturity of the vocal ligament and undifferentiation of the layers of the lamina propria in vocal fold)^(5-7)^. These aspects may generate vocal deviations which are considered common in children and hence they do not indicate the presence of a vocal disorder itself^(8)^. Some degree of breathiness and/or roughness can be expected in children's voices, a factor related to the developmental stage of the child’s body and larynx^(8,9)^.

Devido à complexidade supracitada, é bastante claro na literature que a avaliação vocal deve ser multidimensional. Different factors must be analyzed to determine the presence or absence of a vocal disorder, such as: the parents' complaint and assessment^(9,10)^, child's self-assessment^(11)^, auditory-perceptual and acoustic evaluation of the voice^(2,3,9,12)^, and laryngeal assessment^(13)^. These assessments must be complementary and analyzed together for clinical decision-making, as the relationship between them is not always direct^(6,8)^.

There are still gaps to be filled, specifically, in the relationship between laryngeal assessment and voice assessment. In adults, this relationship is clearer and more consistent^(14)^. However, in the pediatric population, though some studies have shown that children with vocal fold injuries have vocal quality alterations^(15,16)^, other studies have shown that a portion of children with normal larynxes and without vocal complaints also have vocal deviations from the auditory-perceptual point of view^(17,18)^.

The speech tasks used for the auditory-perceptual evaluation in the vocal clinic involve sustained, chained and spontaneous vocal emissions^(19)^. The literature states that we must consider vowel tasks and speak in a complementary way as the vocal characteristics of the same subject may vary depending on the type of task requested^(20)^. For the adult population, sustained emission can be assessed as more deviated in relation to speech tasks^(20)^. However, it raises a question of whether there would be a specific task that could help differentiate healthy voices in children during development, taking all the complexity into consideration, from those associated with lesions in their vocal folds.

Hence, the objective of the present study was to compare the vowel emission and number counting tasks in perceptual-auditory differentiation among children with and without laryngeal lesions.

## METHODS

This is an observational, analytical, transversal, retrospective and prospective study, approved by the institution's ethics committee, under number 2,440,456. Informed consent was waived for the present study as we used a database that referred to a retrospective research, which corresponded to a previous research, in which all the patients had signed the free and informed consent forms. Data were obtained based on the presence or absence of vocal complaints and result of laryngological and vocal evaluation of 70 children, patients from a pediatric service of a reference hospital. The children/parents were invited to undergo a laryngological evaluation and vocal quality. Of the 70 children evaluated, 11 presented vocal fold lesion of behavioral origin and were called group with laryngeal lesion (WLL). In contrast, 59 presented normal laryngological data, without lesions in vocal folds. Of these, we chose to randomize a number that corresponded to three times the number of the other group, for the composition of a control group. Thus, 33 children were selected for the group without laryngeal lesion (WOLL).

The WOLL (n = 33) was consisted of 18 boys and 15 girls, with an average age of 7.07 years (minimum 4 and maximum 10 years), two with vocal complaints; and the WLL (n = 11) was composed of 5 boys and 6 girls, with a mean age of 7.09 years (minimum 4 and maximum 11 years) and diagnosis of lesion in vocal fold(s) of behavioral origin, one with vocal complaint. Regarding the characterization of the WLL, there are three types of lesion/lesion in vocal folds: bilateral vocal nodules (n = 6), epidermoid cyst (n = 2) and diffuse edema (n = 3).

For the selection of data from the previous study for participation in the present study, exclusion criteria were children with: a) glottic clefts not associated with vocal fold lesions, except for posterior (physiological) triangular chinks, b) cold or acute airway obstructions, c) allergic and/or respiratory crisis on the day of collection or in the last 30 days, d) hearing complaint, and e) history of speech-language pathology follow-up for voice disorder.

At the time of the previous study, the laryngological evaluations were performed by a group of physicians, two residents, and one physician and one professor responsible for the service. The results of the evaluation were discussed and issued by consensus. All children went through voice recording.

The vocal material used was comprised of the emission of sustained vowel /é/ and number counting from one to 10, in usual voice and speech. The samples were recorded in a wave sound file, on a Dell® notebook, with an Andrea Pure Audio Interface sound card, unidirectional head microphone, positioned approximately one centimeter from the corner of the participant's mouth in a diagonal position. If there was difficulty in calibration due to the intensity presented by the child (very strong or very weak), the microphone was repositioned in a place where the gain was adequate, which meant sufficient signal level, around two-thirds of the audio window, as indicated in the VOXMETRY® software (CTS Informática, version 2.5).

For the current study, the audio files were edited, disregarding the initial and final portions of the recordings when there is natural instability in the voice, and medium length, lasting between three and four seconds was maintained. Due to the small differences in signal strength during the capturing of voices, the samples were standardized following manual calibration, in the Audacity® program (version 2.0.3).

Regarding cross-sectional data collection, the analysis of vocal material was carried out by a speech-language pathologist (SLP) and voice specialist, with 10 years of clinical experience, master's, doctorate, and professor in the area, who did not previously know the children. The specialist considered the general degree of vocal deviation (G) by means of a numerical scale of three points and considered that 0, 1, 2, and 3 indicated absence of, discrete, moderate, and intense deviations, respectively. In addition, the specialist pointed out whether the child would pass or fail according to the speech material presented (vowel or numbers), in the face of a situation of vocal screening. It was clarified that the evaluator's judgment regarding screening was subjective and they should signal whether in the child, who presented the vocal sample, would be referred to a laryngological evaluation and complete vocal evaluation^(17)^.

Since the aim of the study was to compare the type of voice/speech task in the differentiation of children with and without laryngeal lesion, the specialist initially analyzed the vowel emissions of all children and marked G. Next, they analyzed the number count and made the same marking, without consulting the evaluation for the other task, referring to the same child. Next, they should infer whether, in a situation of vocal screening, the child would pass or fail, considering both the samples. The auditory-perceptual analysis was performed in approximately two hours in a single day, with no interval between the evaluation of the vowel and the numbers. The voices were randomly distributed in the two folders. To analyze the internal consistency of the judge, 20% of the samples were randomly repeated in all tasks (vowel, numbers, and screening).

Next, the Kappa test was performed to identify the internal consistency of the judge. For all tasks, Kappa was higher than 0.6 (vowel 0.62, numbers 0.62, and screening 0.75), which indicated substantial agreement^(21)^. These values, although they could have been closer to 1, which indicated greater agreement, reflected the complexity involved in the auditory-perceptual evaluation of children's voices, due to the process of laryngeal and vocal development. This fact was corroborated by the similar Kappa values obtained in other studies that involved internal agreement of judges in the auditory-perceptual evaluation of the pediatric population^(3,17,22,23)^.

The results were statistically analyzed using Pearson’s Chi-square (>2 groups) and Fisher's exact tests (two groups), which associated two categorical variables for independent groups. An equal proportion test was also used when the occurrence analysis referred to only one of the groups. Statistical analyses were performed in the SPSS® and Statistica®. For all analyses, a significance level of 5% was set.

## RESULTS

It is observed that during the analysis of the overall degree of vocal deviation with sustained vowel task, there was a similar distribution between the groups WOLL and WLL (p = 0.075), with a predominance of mild deviations in both groups. There was a statistically significant difference in the analysis of the overall degree of vocal deviation during the counting task to compare the groups (0.013). WOLL showed a predominance of mild deviations (63.64%), while in WLL there was a predominance of moderate deviations (73.73%) (Table 1). Comparing the WLL and WOLL groups for each degree of vocal deviation, there were differences in the occurrence of grade 3 in the vowel task, being higher in the WLL group (*p*=0.02) and grade 1 in the number task, being higher in the WOLL group (*p*=0.04) (Table 1).

**Table 1:** Association between the overall degree of vocal deviation for the task of sustained vowel and numbers counting and the groups Without Laryngeal Lesion and the With Laryngeal Lesion

Task	Group		Grau do Desvio Vocal				p-value
			0	1	2	3	
Sustained vowel	WOLL	N	2	16	14	1	0.075
		%	6.06	48.48	42.42	3.03	
	WLL	N	0	3	5	3	
		%	0	27.27	45.45	27.27	
Number counting	WOLL	N	4	21	8	0	0.013 *
		%	12.12	63.64	24.24	0	
	WLL		0	3	8	0	
		%	0	27.27	72.73	0	

p < 0.05 – Chi-Squared test; Additional peer analysis: SUSTAINED VOWEL - Degree 0 – WOLL X WLL: p = 0.41; Degree 1- WOLL X WLL: p = 0.38; Degree 2- WOLL X WLL: p = 0.86; Degree 3- 0.02. NUMBER COUNTING - Degree 0 – WOLL X WLL: p = 0.50; Degree 1 – WOLL X WLL: p = 0.04; Degree 2 – WOLL X WLL: p = 0.005; Degree 3- There was no occurrence;

* Statistical test with significance level

**Caption:** N- sample number

For additional analysis, compared to that contained in Table 1, the proportions of each of the judge's response possibilities regarding vocal deviation in sustained vowel were compared for each group. It is observed that in both WOLL and WLL there was a predominance of mild and moderate deviations and there was no difference between them (p=0.81 and p=0.35, respectively). In WOLL, the mild deviations differed from the normal and intense deviations (p=0,04) and showed borderline values ​​when compared to normal voices (p = 0.05). In the WLL, the moderate deviations differed from the normal voices (p = 0.02) and there were no differences when compared to the mild and intense deviations (p = 0.33 for both crossings).

Moreover, as an additional analysis, the proportions of each of the judge's response possibilities regarding vocal deviation during the counting were compared for each group. In the WLL, there were differences in moderate deviations in both the tasks when compared to the mild deviations as well as the normal voices. In WOLL, the mild deviations had differences when compared to the moderate deviations and to the normal voices (p=0.04 and p = 0.03, respectively).

The Table 2 shows that during vocal screening forthe sustained vowel task, there was a similar distribution in the WOLL and WLL groups (p = 0.262). In both groups, most children failed during vocal screening. Regarding the screening situation for the counting task, the percentage of children who failed was higher in the WLL group (81.82%) compared to WOLL (36.36%) (*p* bordering 0.089). We chose to put both the tasks related to vocal screening in groups (sustained vowel and number counting) and compare whether there was any difference between them with regard to the distribution of children who failed in both the tasks. The number of children who passed at least one task was higher in WOLL, while in WLL there was a predominance of children who failed both tasks (p = 0.036).

**Table 2:** Association between the result of vocal screening for the task of sustained vowel, number counting and both tasks and the groups Without Laryngeal Lesion and the With Laryngeal Lesion

Group		Sustained vowel			Number counting			Both tasks		
		Passed	Failed	p-value	Passed	Failed	p-value	Passed	Failed	p-value
WOLL	N	12	21	0.262	21	12	0.08	24	9	0.036^*^
	%	36.36	63.64		63.64	36.36		72.73	27.27	
WLL	N	2	9		2	9		4	7	
	%	18.18	81.82		18.18	81.82		36.36	63.64	

p<0.05 - Fisher's exact test

* Statistical test with significance level

**Caption:** N- sample number

## DISCUSSION

The auditory-perceptual evaluation which is considered the gold standard in the vocal clinic, depends on many aspects, for example, on the experience of the evaluator and his previous training, the task type requested (sustained, chained or spontaneous emission) and the difficulty of consistency internal of the evaluator^(24,25)^.

There are no specific auditory-perceptual evaluation methods for the child population, and so, this type of analysis follows the same parameters as the adult auditory-perceptual evaluation. However, due to the complexity involved in the development of the larynx and voice during childhood^(26)^, the subject deserves further study in the area's literature. Evidence states that breathiness and/or roughness can be considered normal in children's voices^(8)^ and that approximately 65% of the pediatric population has an overall degree of mild or moderate vocal deviation in the auditory-perceptual evaluation, with predominance of the mild degree^(9)^.

In the present study, the task of counting numbers proved to be useful in differentiating children with and without laryngeal lesions. In a research conducted with the adult population, which investigated the influence of the task on the classification of the intensity of dysphonia, it was concluded that sustained vowels are evaluated as more deviated in relation to speech tasks, and vowels are also more susceptible to inter-rater concordance problems^(20)^. The data from the present study show that in children with laryngeal lesions, the task of vowel emission was not adequate to differentiate the two groups, as for both there was a predominance of discrete and (or) moderate deviations. In the task of counting numbers, children with laryngeal lesions were assessed with moderate deviations and children without lesions were assessed with mild deviations.

The fact that children with normal larynx have presented mild and (or) moderate deviations in the vowel emission task reinforces the idea that, even in the absence of lesions, the child population can present vocal deviations of varying degrees. Such results corroborate information brought into study^(9)^, that vocal characteristic of breathiness, instability, and roughness can occur in varying degrees in children's voices and may be related to the process of laryngeal development that the child is going through.

A study analyzed the voices of adolescents aged 13 to 15 years and concluded that sustained vowel was the task capable of identifying the vocal instabilities typical of the vocal change period, not verified in the tasks of counting numbers or reading text^(27)^. The present study shows that for children, the task of sustained vowel follows the same precepts since the laryngeal development process has the consequences of certain types of vocal deviations that are more evident in this type of task.

In addition, on the effect of the selection of speech tasks on inter-rater reliability during the auditory-perceptual evaluation of the voice, a study with 60 dysphonic and non-dysphonic subjects had their samples classified according to the intensity of the deviation by 18 judges. It was concluded that both the number counting and sustained emission tasks were important for the analysis of such reliability^(28)^. As mentioned, in the present study, the number counting task allowed better differentiation between children with and without laryngeal lesion. However, corroborating the aforementioned study, our data indicated that in screening situations children with laryngeal lesion generally tended to fail both in sustained and chained emissions unlike children with normal larynx, who may eventually fail in only one of the tasks. This shows that the complementarity of both continues to have great relevance in the vocal clinic.

In the infant population, there is literature that children with laryngeal lesions have greater perceptual-auditory deviations in sustained vowel tasks, chained speech, and spontaneous speech when compared to a control group^(18)^. However, this type of evaluation is due to the complexity inherent to the vocal deviations common to the laryngeal development process, maintaining a very fine line between what should be considered normal to the process and what can already be considered as a disorder. Hence, having the information that the task of counting numbers may be more appropriate for this differentiation contributes to the practice of the speech therapist in the vocal clinic.

In this view, it is possible to infer that although the complementarity of the two tasks (vowel emission and number counting) remains fundamental in the process of vocal evaluation of children, special attention is directed to chained speech. This can provide more information when there are doubts regarding deviations common to the process of child development and deviations indicative of possible disorders.

As for limitations of this study, the reduced number of children diagnosed with laryngeal lesion was mentioned. Hence, studies that increase the number of subjects in this condition are necessary to generalize the data to other realities so they can be considered during the performance of vocal screenings in global actions. It is envisaged that subsequent studies can ratify the information that samples of chained speech are the most indicated in the auditory-perceptual evaluation of the voice of the infant population in screening situations.

## CONCLUSION

The results indicate that the task of counting numbers contributes to the auditory-perceptual differentiation of children with and without laryngeal lesions, as it presents with more intense deviations in children with a lesion. The sustained vowel, on the other hand, presents itself in a similar way between these two groups, with a predominance of mild and (or) moderate deviations in both, and, therefore, it was not useful to differentiate them. In a situation of vocal screening, children with laryngeal lesions fail both in sustained vowel and number counting, unlike children without lesion, who commonly fail in only one of the tasks.
