# Supplementary material for: Auditory-perceptual evaluation of voice: comparing different speech tasks to identify children with and without laryngeal lesions
Source: Codas. 2023 Mar 3;35(2):e20210198. doi: 10.1590/2317-1782/20212021198en (PMC10010427; doi:10.1590/2317-1782/20212021198en)
Supplement: Translation

# Translation: Avaliação perceptivo-auditiva da voz: comparação de diferentes tarefas de fala na identificação de crianças com e sem lesões laríngeas

**DOI:** 10.1590/2317-1782/20212021198pt

**Authors:** Cintia Conceição Costa, Ana Paula Dassie Leite, Glaucya Madazio, Mara Behlau

**Affiliations:** 1 Departamento de Fonoaudiologia, Universidade Estadual do Centro-Oeste – UNICENTRO - Irati (PR), Brasil.; 2 Centro de Estudos da Voz – CEV - São Paulo (SP), Brasil.

**Keywords:** Qualidade Vocal, Disfonia, Crianças, Voz, Distúrbios da Voz

## Abstract

**Objetivo:**

comparar as tarefas de emissão de vogal e de contagem de números na diferenciação perceptivo-auditiva de crianças com e sem lesão laríngea.

**Método:**

Estudo observacional, analítico, transversal. Utilizou-se banco de dados de uma pesquisa de doutorado, com resultados de avaliações laringológicas e gravação de amostras vocais de 44 crianças que se dividiram em: Grupo sem lesão laríngea (GSLL), com 33 crianças; e grupo com lesão laríngea (GCLL), com 11 crianças. Para a avaliação perceptivo-auditiva, as amostras vocais foram separadas de acordo com o tipo de tarefa e analisadas separadamente por uma juíza, que analisou o grau geral do desvio vocal e informou se, diante de uma situação de triagem, a criança passaria ou falharia.

**Resultados:**

Houve diferença entre os grupos GSLL e GCLL quanto ao grau geral do desvio vocal para tarefa de emissão de números, com predomínio de desvios discretos no GSLL e moderados no GCLL. Quanto à triagem, houve diferença entre os grupos para a tarefa de contagem, com mais falhas no GCLL. Os grupos foram semelhantes na tarefa de vogal, tanto no que se refere à intensidade do desvio quanto ao resultado da triagem. A maior parte das crianças do GCLL falhou em ambas as tarefas na situação de triagem vocal, com diferença em relação às crianças do GSLL que, em geral, falharam em apenas uma tarefa.

**Conclusão:**

A tarefa de contagem de números contribui para a diferenciação auditiva de crianças com e sem lesão laríngea, por identificar desvios de maior intensidade em crianças com lesão.

## INTRODUÇÃO

Os distúrbios vocais na infância podem impactar de forma negativa a vida da criança em relação à comunicação eficiente e à participação nas atividades sociais, educacionais e em grupo^(1)^. Embora a ocorrência das alterações vocais na criança varie de 6 a 38%^(2,3)^, muitas vezes os sintomas vocais na infância são negligenciados pela família, que não entende a rouquidão como um problema, e que valoriza, em muito maior proporção, somente as alterações de fala dos filhos^(4)^.

Há uma grande complexidade na avaliação das vozes infantis, principalmente pelo fato de que as crianças apresentam características anatomofisiológicas muito diferentes das dos adultos, tanto relacionadas à respiração (pressão subglótica superior à necessária para a fonação, causando desajuste entre os sistemas), quanto relacionada às características laríngeas (imaturidade do ligamento vocal e indiferenciação das camadas da lâmina própria da prega vocal^(5-7)^. Tais aspectos podem gerar desvios vocais considerados comuns às crianças, ou seja, que não indicam a presença de um distúrbio propriamente dito^(8)^. Estima-se, por exemplo, que algum grau de soprosidade e/ou rugosidade pode ser considerado esperado nas vozes infantis, fator que está relacionado à fase de desenvolvimento corporal e laríngeo que a criança se encontra^(8,9)^.

Devido à complexidade supracitada, é bastante claro na literatura que a avaliação vocal deve ser multidimensional. Diferentes fatores devem ser analisados para determinar a presença ou a ausência de um distúrbio vocal, tais como: a queixa e a avaliação dos pais^(9,10)^, a autoavaliação da criança^(11)^, a avaliação perceptivo-auditiva e acústica da voz^(2,3,9,12)^, e a avaliação laringológica^(13)^. Tais avaliações devem ser complementares e analisadas em conjunto para a tomada de decisão clínica, uma vez que nem sempre a relação entre elas é direta^(6,8)^.

Particularmente quanto à relação entre a avaliação laringológica e a avaliação da voz, ainda há lacunas a serem preenchidas. Em indivíduos adultos, essa relação já é mais clara e consistente^(14)^. No entanto, na população infantil, embora alguns estudos comprovem que crianças com lesões em pregas vocais apresentam alterações perceptivo-auditivas da voz^(15,16)^, outras pesquisas demonstram que uma parcela de crianças com laringes normais e sem queixas também apresentam desvios vocais do ponto de vista perceptivo-auditivo^(17,18)^.

As tarefas de fala utilizadas para a avaliação perceptivo-auditiva na clínica vocal envolvem emissões sustentadas, encadeadas e espontâneas^(19)^. A literatura afirma que devemos considerar as tarefas de vogal e fala de forma complementar, uma vez que as características vocais de um mesmo sujeito podem variar a depender do tipo de tarefa solicitada^(20)^. Para a população adulta, a emissão sustentada pode ser avaliada como mais desviada em relação às tarefas de fala^(20)^. No entanto, questiona-se, aqui, se para vozes infantis, considerando toda a sua complexidade, haveria alguma tarefa, em especial, que poderia contribuir na diferenciação de vozes saudáveis, em processo de desenvolvimento, daquelas associadas a lesões em pregas vocais.

Diante o exposto, o objetivo do presente estudo foi comparar as tarefas de emissão de vogal e de contagem de números na diferenciação perceptivo-auditiva de crianças com e sem lesão laríngea.

## MÉTODO

Trata-se de trabalho observacional, analítico e transversal, aprovado pelo comitê de ética da instituição, sob o número 2.440.456. O termo de consentimento livre e esclarecido foi dispensado para a presente pesquisa pois utilizou-se banco de dados referente à pesquisa retrospectiva correspondente a uma pesquisa anterior, na qual todos os pacientes assinaram o termo de consentimento livre e esclarecido. Foram obtidos os dados sobre a presença ou ausência de queixa vocal e resultado da avaliação laringológica e vocal de 70 crianças, pacientes de um serviço de pediatria de um hospital de referência. Na ocasião, as crianças/responsáveis eram convidadas a passar por avaliação laringológica e da qualidade vocal. Das 70 crianças avaliadas na ocasião, 11 apresentaram lesão em prega vocal de origem comportamental e aqui compõem o grupo denominado Com Lesão Laríngea (GCLL). Ainda do total, 59 apresentaram dados laringológicos considerados normais, sem lesões em pregas vocais e, destas, optou-se por randomizar um número correspondente a três vezes o número do outro grupo, para a composição de um grupo controle. Desta forma, foram selecionadas 33 crianças para compor o grupo denominado Sem Lesão Laríngea (GSLL).

O GSLL (n = 33) foi composto por 18 meninos e 15 meninas, com média de idade de 7,07 anos (mínimo 4 e máximo 10 anos), sendo duas com queixa vocal do responsável; e o GCLL (n = 11) foi composto por 5 meninos e 6 meninas, com média de idade de 7,09 anos (mínimo 4 e máximo 11 anos) e diagnóstico de lesão e (ou) alteração (ões) em prega (s) vocal (is) de origem comportamental, sendo uma delas com queixa vocal do responsável. Quanto a caracterização do GCLL, no que se refere ao tipo de lesão/alteração em pregas vocais, tem-se: nódulos vocais bilaterais (n=6), cisto epidermóide (n=2) e edema difuso (n=3).

Para a seleção dos dados do estudo anterior para a participação na presente pesquisa, foram considerados critérios de exclusão para ambos os grupos: crianças com fendas glóticas não associadas a lesões em pregas vocais, exceto fendas triangulares posteriores (fisiológicas); gripes, resfriados ou obstruções agudas de vias aéreas por quaisquer motivos no dia da coleta; crises alérgicas e/ou respiratórias no dia da coleta ou nos últimos trinta dias; queixa auditiva; histórico de acompanhamento fonoaudiológico para distúrbio de voz.

As avaliações laringológicas, na ocasião do estudo anterior, foram realizadas por um grupo de médicos otorrinolaringologistas, sendo dois residentes, um médico responsável pelo serviço e um professor responsável pelo serviço. Os resultados da avaliação foram discutidos e emitidos por consenso. Todas as crianças participantes passaram por gravação da voz.

O material vocal utilizado compreendeu a emissão da vogal sustentada “é” e contagem de números de um a 10, em voz e fala habituais. As amostras foram gravadas em arquivo de som wave, em notebook da marca Dell®, com microfone unidirecional de cabeça, da marca Karsect, modelo HT-9, acoplado à interface de som Andrea Pure Áudio. O microfone foi posicionado a aproximadamente um centímetro do canto da boca do participante (posição diagonal). Caso houvesse dificuldade de calibração devido à intensidade apresentada pela criança (muito forte ou muito fraca), o microfone era reposicionado em local onde o ganho fosse adequado, o que significa nível suficiente de sinal, ao redor de dois terços da janela de áudio, como indicado no programa VOXMETRIA® (CTS Informática, versão 2.5).

Para a edição dos arquivos de áudio, foram desprezados trechos iniciais e finais das gravações, que geralmente correspondem a um período de instabilidade natural da voz, mantendo-se o trecho médio, com duração entre três e quatro segundos. Devido às pequenas diferenças na intensidade do sinal durante a captação das vozes, as amostras foram padronizadas seguindo calibração manual, no programa Audacity® (versão 2.0.3).

No que se refere à coleta transversal de dados, as análises do material vocal foram realizadas por uma juíza fonoaudióloga especialista em voz, com 10 anos de experiência clínica, mestrado, doutorado e docente da área, que não conhecia previamente as amostras, e que considerou o grau geral de desvio vocal (G) por meio de escala numérica de três pontos, considerando que 0 indicava ausência de desvio, 1 desvio discreto, 2 desvio moderado e 3 desvio intenso. Além disso, a juíza assinalou se, diante de uma situação de triagem vocal, a criança passaria ou falharia segundo o material de fala apresentado (vogal ou números). Esclarece-se que o julgamento do avaliador quanto à triagem foi subjetivo e ele deveria sinalizar se diante de uma criança que apresentasse tal amostra vocal, a encaminharia para avaliação laringológica e avaliação vocal completa^(17)^.

Uma vez que o objetivo do estudo foi comparar o tipo de tarefa de voz/fala na diferenciação de crianças com e sem lesão laríngea, a juíza analisou, inicialmente, as emissões de vogais de todas as crianças, fazendo a marcação do G. Na sequência, analisou a contagem de números e fez a mesma marcação, sem consultar sua avaliação para a outra tarefa, referente à mesma criança. Em seguida, a juíza deveria inferir se, em uma situação de triagem vocal, a criança passaria ou falharia, considerando ambas as amostras. A análise perceptivo-auditiva foi realizada em aproximadamente duas horas em um único dia, não havendo intervalo entre a avaliação da vogal e dos números. As vozes foram distribuídas aleatoriamente nas duas pastas. Para análise da consistência interna da juiza, foram repetidas 20% das amostras, de modo aleatório, em todas as tarefas (vogal, números e triagem).

Em seguida, foi realizado o teste de Kappa a fim de identificar a consistência interna da juíza. Para todas as tarefas, o Kappa foi superior a 0,6 (vogal 0,62; números 0,62 e triagem 0,75), indicando concordância substancial^(21)^. Tais valores, embora pudessem ter sido mais próximos de 1, indicando maior concordância, refletem a complexidade envolvida na avaliação perceptivo-auditiva de vozes infantis, devido ao processo de desenvolvimento laríngeo e vocal. Tal fato é corroborado pelos valores semelhantes de Kappa obtidos em outros estudos envolvendo concordância interna dos de juízes na avaliação perceptivo-auditiva da população pediátrica^(3,17,22,23)^.

Os resultados foram analisados estatisticamente por meio dos testes Qui-quadrado de Pearson (>2 grupos) e Exato de Fisher (2 grupos), que associam duas variáveis categóricas para grupos independentes. Também foi utilizado teste de igualdade de proporções, quando a análise de ocorrência se referia a apenas um dos grupos. As análises estatísticas foram feitas nos programas SPSS® e Statistica®. Para todas as análises, foi adotado nível de significância de 5%.

## RESULTADOS

Observa-se que, na análise do grau geral de desvio vocal com a tarefa de emissão de vogal, houve distribuição semelhante entre os grupos GSLL e GCLL (p = 0,075), com predomínio de desvios considerados discretos e moderados em ambos os grupos. Houve diferença estatisticamente significante na análise do grau geral de desvio vocal com a tarefa de contagem de números para comparação dos grupos (p = 0,013). O GSLL apresentou predomínio de desvios considerados discretos (63,64%), enquanto no GCLL houve predomínio de desvios considerados moderados (73,73%) (Tabela 1). Comparando-se os grupos GSLL e GCLL para cada grau de desvio vocal, houve diferença entre eles apenas na ocorrência de grau 3 na tarefa de vogal, sendo ela maior no GCLL (p=0,02) e na ocorrência de grau 1 na tarefa de números, sendo ela maior no GSLL (p=0,04) (Tabela 1).

**Tabela 1:** Associação entre o grau geral de desvio vocal para a tarefa de emissão de vogal e números e os grupos Grupo Sem Lesão Laríngea e Grupo Com Lesão Laríngea

Tarefa	Grupo		Grau do Desvio Vocal				p-valor
			0	1	2	3	
Vogal	GSLL	N	2	16	14	1	0,075
		%	6,06	48,48	42,42	3,03	
	GCLL	N	0	3	5	3	
		%	0	27,27	45,45	27,27	
Números	GSLL	N	4	21	8	0	0,013*
		%	12,12	63,64	24,24	0	
	GCLL	N	0	3	8	0	
		%	0	27,27	72,73	0	

p<0,05 – Qui-Quadrado de Pearson; Comparações aos pares – Teste de igualdade de proporções: VOGAL - Grau 0 – GSL X GCL: p = 0,41; Grau 1- GSL X GCL: p = 0,38; Grau 2- GSL X GCL: p = 0,86; Grau 3- 0,02. NÚMEROS - Grau 0 – GSL X GCL: p = 0,50; Grau 1 – GSL X GCL: p = 0,04; Grau 2 – GSL X GCL: p = 0,005; Grau 3- Não houve ocorrência

* Teste estatístico com nível de significância

**Legenda:** N- número de participantes

Como análise adicional em relação ao contido na tabela 1, as proporções de cada uma das possibilidades de resposta da juíza quanto ao desvio vocal na emissão de vogal foram comparadas, aos pares, para cada um dos grupos de forma independente. Tanto no GSLL quanto no GCLL houve predomínio de desvios discretos e moderados, sem diferença entre eles (p=0,81 e p=35, respectivamente). No GSLL, os desvios discretos diferenciaram-se dos desvios dentro da variabilidade normal e intensos (p=0,01 e p=0,003, respectivamente) e os desvios moderados diferenciaram-se dos desvios intensos (p=0,04) e tiveram valores limítrofes quando comparados aos desvios dentro da variabilidade normal (p=0,05). No GCLL, os desvios moderados diferenciaram-se dos desvios dentro da variabilidade normal (p =0,02) e não tiveram diferenças quando comparados aos desvios discretos e intensos (p=0,33 para ambos os cruzamentos).

Foi realizada ainda, como análise adicional, as proporções de cada uma das possibilidades de resposta da juíza quanto ao desvio vocal na emissão de números foram comparadas, para cada um dos grupos de forma independente. No GCLL os desvios moderados tiveram diferenças tanto quando comparados aos desvios dentro da variabilidade normal e discretos. No GSLL os desvios discretos tiveram diferenças tanto quando comparados aos desvios moderados quanto aos desvios considerados dentro da variabilidade normal (p=0,04 e p = 0,03, respectivamente).

A Tabela 2 mostra que na situação de triagem vocal para a tarefa de emissão de vogal, houve distribuição semelhante nos grupos GSLL e GCLL (p=0,262). Em ambos os grupos, a maioria das crianças falhou na triagem. No que se refere à situação de triagem para a tarefa de contagem, a porcentagem de crianças que falhou foi maior no GCLL (81.82%), quando comparadas ao GSLL (36.36%) (p limítrofe de 0,089). Optou-se por agrupar ambas as tarefas relacionadas à triagem vocal (emissão de vogal e de contagem de números) e comparar se haveria diferença entre os grupos quanto à distribuição de crianças que falharam em ambas as tarefas. O número de crianças que passou em pelo menos uma tarefa foi maior no GSLL, enquanto no GCLL houve predomínio de crianças que falharam em ambas as tarefas (p=0,036) (Tabela 2).

**Tabela 2:** Associação entre o resultado da triagem vocal para a tarefa de emissão de vogal, números e geral e os grupos Grupo Sem Lesão Laríngea e Grupo Com Lesão Laríngea

Grupo		Vogal			Números			Geral		
		Passou	Falhou	p-valor	Passou	Falhou	p-valor	Passou	Falhou	p-valor
GSLL	N	12	21	0,262	21	12	0,08	24	9	0,036^*^
	%	36,36	63,64		63,64	36,36		72,73	27,27	
GCLL	N	2	9		2	9		4	7	
	%	18,18	81,82		18,18	81,82		36,36	63,64	

p <0,05 – Teste Exato de Fisher

* Teste estatístico com nível de significância

**Legenda:** N- número de participantes

## DISCUSSÃO

A avaliação perceptivo-auditiva, embora considerada padrão-ouro na clínica vocal, depende de muitos aspectos, relacionados por exemplo à experiência do avaliador e seu treinamento anterior, o tipo de tarefa solicitada (emissão sustentada, encadeada ou espontânea) e da dificuldade de consistência interna de quem avalia^(24,25)^.

Não há métodos específicos de avaliação perceptivo-auditiva para a população infantil e, por isso, esse tipo de análise segue os mesmos parâmetros da avaliação perceptivo-auditiva do adulto. No entanto, devido à complexidade envolvida no desenvolvimento da laringe e da voz durante a infância^(26)^, o assunto merece maior aprofundamento na literatura da área. Encontram-se evidências de que soprosidade e/ou rugosidade podem ser consideradas normais nas vozes infantis^(8)^ e que aproximadamente 65% da população pediátrica apresenta grau geral de desvio vocal discreto ou moderado na avaliação perceptivo-auditiva, com predomínios do grau discreto^(9)^.

No presente estudo, pôde-se observar que a tarefa de contagem de números diferenciou os grupos de crianças com laringe normal e com lesão laríngea. Em pesquisa realizada com a população adulta, que investigou a influência da tarefa na classificação da intensidade da disfonia, concluiu-se que as vogais sustentadas são avaliadas como mais desviadas em relação às tarefas de fala, sendo as vogais também mais suscetíveis aos problemas de confiabilidade interavaliadores^(20)^. Os dados do presente estudo mostram que em crianças com lesão de laringe, a tarefa de emissão de vogal não se mostrou adequada para diferenciar os dois grupos, uma vez que para ambos houve predomínio de desvios discretos e (ou) moderados. Já na tarefa de contagem de números, crianças com lesão de laringe foram avaliadas com desvios moderados e, crianças sem lesão foram avaliadas com desvios discretos.

O fato de crianças com laringe normal terem apresentado desvios discretos e (ou) moderados na tarefa de emissão de vogal reforça a ideia de que, mesmo na ausência de distúrbio, a população infantil pode apresentar desvios vocais de graus bastante variados. Tais resultados corroboram informações trazidas em estudo^(9)^, de que características vocais de soprosidade, instabilidade e rugosidade podem acontecer em graus variados nas vozes infantis e podem estar relacionadas ao processo de desenvolvimento laríngeo pelo qual a criança está passando.

Um estudo que analisou vozes de adolescentes de 13 a 15 anos concluiu que a vogal sustentada é a tarefa capaz de identificar as instabilidades vocais típicas do período de muda vocal, não verificadas nas tarefas de contagem de números ou leitura de texto^(27)^. Os dados do presente estudo mostram que para as crianças da população geral, a tarefa de emissão sustentada segue os mesmos preceitos, uma vez que o processo de desenvolvimento laríngeo tem como consequências determinados tipos de desvios vocais que são mais evidentes nesse tipo de tarefa.

Ainda sobre o efeito da seleção de tarefas de fala na confiabilidade interavaliadores durante a avaliação perceptivo-auditiva da voz, um estudo com 60 sujeitos disfônicos e não disfônicos tiveram suas amostras classificadas quanto à intensidade do desvio, por 18 juízes. Concluiu-se que tanto a tarefa de contagem de números quanto a tarefa de emissão sustentada são importantes para a análise de tal confiabilidade^(28)^. Conforme mencionado, no presente estudo a tarefa de contagem de números possibilitou melhor diferenciação entre crianças com e sem lesão laríngea. No entanto, corroborando o estudo supracitado, os dados aqui obtidos indicam que, em geral, em situações de triagem crianças com lesão laríngea tendem a falhar tanto em emissões sustentadas quanto encadeadas, diferentemente de crianças com laringe normal, que podem eventualmente falhar em apenas uma das tarefas. Isso mostra que a complementariedade de ambas continua tendo grande relevância na clínica vocal.

Na população infantil, embora também já haja na literatura a informação de que crianças com lesões laríngeas apresentam maiores desvios perceptivo-auditivos nas tarefas de vogal sustentada, fala encadeada e fala espontânea quando comparadas a um grupo controle^(18)^, este tipo de avaliação se depara com a complexidade inerente aos desvios vocais comuns ao processo de desenvolvimento laríngeo, mantendo linha bastante tênue entre o que deve ser considerado normal ao processo e o que já pode considerado como distúrbio. Nesse sentido, ter a informação de que a tarefa de contagem de números pode ser mais adequada para essa diferenciação, contribui para a prática do fonoaudiólogo na clínica vocal.

Diante do exposto, é possível inferir que embora a complementariedade das duas tarefas (emissão de vogal e contagem de números) mantém-se como fundamental no processo de avaliação vocal das crianças, direciona-se atenção especial à fala encadeada, que pode fornecer mais informações quando há dúvidas sobre desvios comuns ao processo de desenvolvimento infantil e desvios indicativos de possíveis distúrbios.

Como limitações deste estudo, menciona-se o número reduzido de crianças com diagnóstico de lesão laríngea. Nesse sentido, estudos que ampliem o número de sujeitos nessa condição são necessários para que seja possível a generalização dos dados para outras realidades e, assim, eles possam ser considerados durante a realização de triagens vocais em ações globais. Vislumbra-se que estudos subsequentes possam ratificar a informação de que amostras de fala encadeada sejam as mais indicadas na avaliação perceptivo-auditiva da voz da população infantil em situações de rastreio.

## CONCLUSÃO

Os resultados indicam que a tarefa de contagem de números contribuiu para a diferenciação auditiva de crianças com e sem lesão laríngea, por se apresentar com desvios de maior intensidade em crianças com lesão. Já a emissão sustentada de vogais se apresentou de forma semelhante entre esses dois grupos, com predomínio de desvios discretos e (ou) moderados em ambos e, portanto, não se mostrou útil para diferenciá-los. Em situação de triagem vocal, crianças com lesão laríngea falharam tanto na emissão sustentada quanto encadeada, diferentemente das crianças sem lesão, que comumente falharam em apenas uma das tarefas.
